# Function of the tail in myliobatid rays: role in controlling body stability

**DOI:** 10.1098/rsos.251269

**Published:** 2025-11-05

**Authors:** Júlia Chaumel, Connor White, George Lauder

**Affiliations:** ^1^ Organismic and Evolutionary Biology, Harvard University, Cambridge, MA, USA; ^2^ Department of Environmental Sciences, University of Virginia, Charlottesville, VA, USA

**Keywords:** stingray, myliobatidae, tail, hydrodynamics, stability, locomotion

## Abstract

Eagle rays, cownose rays and manta rays are the only batoid families exhibiting oscillatory locomotion, and are characterized by long, slender tails. This study investigates whether tail length influences body stability when the pectoral fins are held in an extended, static position. We measured relative tail lengths across the four families (Rhinopteridae, Myliobatidae, Aetobatidae and Mobulidae), finding that spotted eagle rays have the longest tails (greater than 4× body length (BL)), while giant manta rays have the shortest (approx. 0.9× BL). To test the effects of tail length on posture and stability, we used three-dimensional-printed models based on a myliobatid body and a NACA 0012 foil in a flow tank across increasing speeds. Pitch, roll and overall dynamic body acceleration (ODBA) were recorded using embedded accelerometers. Models without tails showed increased roll and ODBA, while models with tails greater than or equal to 0.9× BL, which was the minimum length found in measured animals, maintained a steadier position. This result indicates that tails enhance passive stability by providing drag-based damping and a restoring torque that helps the ray models resist and recover from disturbances. As longer tails did not further improve stability, tails exceeding 0.9× BL may serve additional roles, such as communication, mating or sensing.

## 1. Introduction

Elasmobranchs are a group of cartilaginous fishes that includes sharks and batoids (rays and skates). Although elasmobranchs comprise only approximately 1500 species, they exhibit a great diversity of body shapes, which are reflective of functional variation in how these groups swim [1–3]. Sharks typically have fusiform bodies and a heterocercal caudal fin, which oscillates laterally to generate thrust and propel the animal through the water [2,4,5]. In contrast, batoids are dorsoventrally flattened with expanded pectoral fins. Although basal batoids (e.g. guitarfishes, electric and shovelnose rays) use their tails for propulsion, most batoids generate thrust using their pectoral fins, enabling the tails to perform a variety of functions that differ across species [1,6,7].

Batoid pectoral fin kinematics can be divided into two basic types: undulatory, where waves propagate backward along the pectoral fins, and oscillatory, where the entire pectoral fin moves vertically in unison with a high amplitude (similar to flying birds) [1,6,8–11]. Most batoids—including skates and stingrays such as dasyatids—swim by undulatory locomotion defined by multiple travelling waves along the antero-posterior orientation of the fins, which is correlated with low aspect ratio (AR) bodies (diamond or rounder shapes) [1,6]. These batoids are mainly benthic, as undulatory locomotion is considered to provide high degree of manoeuvrability [6,12]. In contrast, oscillatory locomotion is less common, as it is only performed by species from four families of the order Myliobatiformes: Myliobatidae (eagle rays), Aetobatidae (pelagic eagle rays), Rhinopteridae (cownose rays) and Mobulidae (manta rays) [7,13]. Species in these families will be referred to here as myliobatids. As oscillatory batoids, myliobatids present triangular body shapes characterized by high ARs (wingspan greater than the length) [6,12,14]. Oscillatory locomotion with high AR fins increases locomotor efficiency and is reflected in myliobatid species’ ability for sustained as well as high-speed swimming, allowing for long distance migration and extensive vertical movements, features visible in a more pelagic lifestyle than other batoids [8,10,14–17].

Due to their morphology and corresponding locomotory performance, myliobatids have been a focal point for bioinspired design and extensively studied in fields such as robotics, mechanical engineering, fluid dynamics and biomechanics [10,12,18–22]. These studies have primarily examined how pectoral fins generate thrust and how variations in body morphology influence locomotor efficiency. However, one defining feature of myliobatid rays has been largely overlooked: a slender, whip-like tail with a tapered morphology that can extend up to four times the animal’s body length (BL) [7,23]. This tail is a relatively stiff structure formed by fused vertebrae (the caudal synarcual), which minimizes bending along the tail length, and maintains the tail in a relatively straight, trailing position behind the animal during both active locomotion and gliding (electronic supplementary material, videos S1 and S2) [23].

The presence of a long, fused vertebral structure within the elongated and tapered tail is unusual among vertebrates. Tapered tails are usually formed by a segmented vertebral column, which allows for a greater range of vertical and horizontal motion [24]. In aquatic locomotion, tapered tails with relatively low vertical surface area are not efficient at generating thrust, as this morphology limits the tail’s ability to displace water [25]. Instead, tapered tails serve other functions such as providing momentum during swimming, counterbalancing, sensing or defence [25–28]. Similar to other aquatic vertebrates, several batoids also possess long and tapered tails with segmented vertebrae [7]. Although these tails have not been extensively studied, some functions, such as propulsion and defence, can be inferred from associated structures like fins linked to the tails in several species, and a barb in the case of batoids from the order Myliobatiformes [7,29,30]. However, the non-segmented, rigid structure, tapered morphology and the absence of fins on the myliobatid’s tail suggest that these tails may have alternative functions. Recent evidence indicates that the myliobatid tail serves a mechanosensory function, acting like a hydrodynamic antenna, detecting water movements potentially generated by surrounding prey, predators or conspecifics [23]. However, to date, no studies have explored whether the elongated tail in myliobatids plays any role in influencing locomotion or in providing stability for the moving body.

In this project, we experimentally investigate the hydrodynamic effects of long and slender tails on myliobatid body stability using physical models. Many fishes expend a considerable amount of energy to maintain stability during locomotion and gliding, and morphological adaptations that passively contribute to stability are a common feature [31–34]. We hypothesize that tails act as passive stabilizers, similar to the stabilizing effect of long, slender tails attached to aerial kites [35–37]. Our analysis of how tail length influences body stability is confined to ray-based models in a static configuration with laterally expanded pectoral fins held in position.

To understand how tails vary in morphology across myliobatids and to provide data on which to base the experimental plan, we measured tail and body size across several species of the four oscillatory myliobatid families and identified scaling relationships between tail and body morphology. Next, to quantify how tail lengths affected stability (defined as the degree of movement over time), we examined the effects of three different tail lengths on roll, pitch and overall dynamic body acceleration (ODBA). To measure these variables, we used accelerometers placed inside a three-dimensional-printed myliobatid body model, which was towed in a recirculating flow tank, at increasing flow speeds. Finally, as the pectoral fin and body of myliobatid rays are similar in profile to an aerofoil [8,12], we repeated these experiments using a body model based on a NACA 0012 aerofoil to assess the stability of a foil shape relative to a body shape of a myliobatid ray. NACA 0012 foils are teardrop-shaped, symmetric foils with no camber, known for their high lift-to-drag ratio and low drag coefficient, and are commonly used in aerodynamic studies and experimental and computational analyses of aquatic propulsion [5,38–43]. While our models were tethered, which altered the force distribution relative to free-swimming animals, they enable controlled, repeatable comparisons demonstrating how tail length influences postural stability in both the myliobatid model and NACA 0012 model.

Our results suggest a functional relationship between experimental results and biology, as the minimum tail length required to stabilize the models (0.9× BL) agrees with the shortest tail length observed in myliobatids. This study offers a starting point for understanding the hydrodynamic role of the tail in myliobatid rays and lays the groundwork for future investigations into the evolution and function of tails in body stability during aquatic locomotion. Finally, these results can offer a source of inspiration for the design of robotic devices based on myliobatid rays, in addition to offering new insights into how these species interact with their environment.

## 2. Material and methods

### 2.1. Animal morphometrics and density

**Figure 1.**
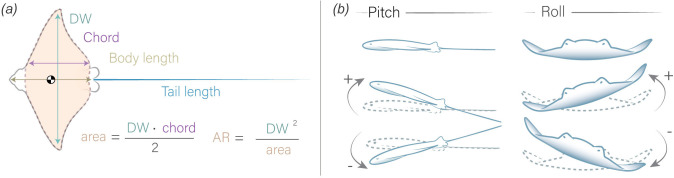
Schematic representation of the measured morphometric variables and ray movement patterns. (*a*) Morphometric variables measured and calculated from specimens, including DW (distance between the tips of the pectoral fins), chord length (distance between the dorsal and ventral points at the base of a pectoral fin), BL (distance from the head to the base of the tail) and tail length (distance from the base to the tip of the tail). The total body area (excluding the head, lobes and pelvic fins) was also measured, along with the AR of the pectoral fins. The centre of mass (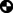) is located anterior to the pectoral fin tips, as described by [6]. (*b*) Schematic representation of the myliobatid model’s movements, illustrating positive pitch (upward orientation) and negative pitch (downward orientation), positive roll (tilt to the right) and negative roll (tilt to the left).

For morphometric analysis, we measured a total of 76 myliobatids from the four different oscillatory families: 9 from Aetobatidae (*Aetobatus narinari* = 9)*,* 42 from Myliobatidae (*Myliobatis californica* = 29; *My. freminvillei* = 3; *My. goodei* = 3; *My. australis* = 2; *My. aquila* = 1; *Aetomylaeus nichofii* = 2; *A. maculatus* = 1; *A. bovinus* = 1); 8 from Rhinopteridae (*Rhinoptera bonasus =* 6; *R. brasiliensis* = 2) and 17 from Mobulidae (*Mobula mobular* = 5; *Mo. thurstoni* = 6; *Mo. kuhlii* = 4; *Mo. birostris* = 2) (electronic supplementary material, table S1). The specimens were preserved in 70% ethanol and obtained from several ichthyology collections, including the Harvard Museum of Comparative Zoology (MCZ), Texas A&M University (TCWC) and The Natural History Museum of London (NHM). We measured most mobulids (family Mobulidae) from photographs shared by CSIRO’s Australian National Fish Collection in Hobart, Australia. We took dorsal images for each animal, ensuring that the tail was fully extended and clearly visible alongside a scale for accurate measurements and a label to indicate the sex. Embryos and specimens with broken or missing tails were excluded from the study. We analysed each image in ImageJ software (National Institutes of Health, Bethesda, MD, USA) to measure disc width (DW, the distance between the tips of the pectoral fins), body length (BL, the distance from the snout to the base of the tail, between the pelvic fins), tail length (the distance from the base of the tail to the tail tip) and pectoral fin chord (the length of the pectoral fin base) (figure 1). Using these measurements, we calculated additional indices such as the tail length-to-BL ratio, tail length-to-DW ratio, estimated body area (DW × chord/2; which did not include the head, lobes and pelvic fins and the pectoral fins are assumed to be triangles) and the AR (DW^2^/area) (figure 1).

We calculated the body density of 34 individuals of a total of nine myliobatid species from the Harvard MCZ ichthyology collection (electronic supplementary material, table S2). To determine their density, we measured the weight of the specimens in air (*W*_a_) and in liquid (*W*_l_). To measure the weight in liquid, we suspended the specimens in a bucket filled with 70% ethanol using a balance, ensuring that the specimens did not touch the bottom or sides of the bucket. Based on Archimedes’ principle, the difference between *W*_a_ and *W*_l_ corresponds to the weight of the displaced fluid (buoyant force) [44]. Since the specimens were preserved in 70% ethanol (density = 0.881 g ml^−1^), the water in their tissues had been replaced by 70% ethanol, resulting in absolute densities less than 1 g ml^−1^ (the density of water). Assuming that the specimens’ bodies are homogeneous, that the volume of the animal did not change during preservation and that the water of their body is fully replaced by ethanol, we assumed that the weight of the preserved ray in ethanol would be the same as the weight of a non-preserved ray in water. As the density of water to be 1 g ml^−1^, we calculated the density of the ray using equation (2.1).


(2.1) $$\rho_{ray}=\frac{W_{a}}{W_{a}-W_{l}}\;\times \;\rho_{water}.$$

This assumption introduces a small systematic error, as our measurements were conducted in 70% ethanol. However, this simplification facilitates comparison with previously reported density values and provides an approximate density value across different myliobatid species to ensure the density of our models is appropriate. Once the difference between the density of water and density of ethanol are accounted for, the estimated densities of individuals ranged from 1.04 to 1.12 g ml^−1^ and averaged 1.08 ± 0.01 g ml^−1^ across all measured species.

### 2.2. Model design and preparation

**Figure 2.**
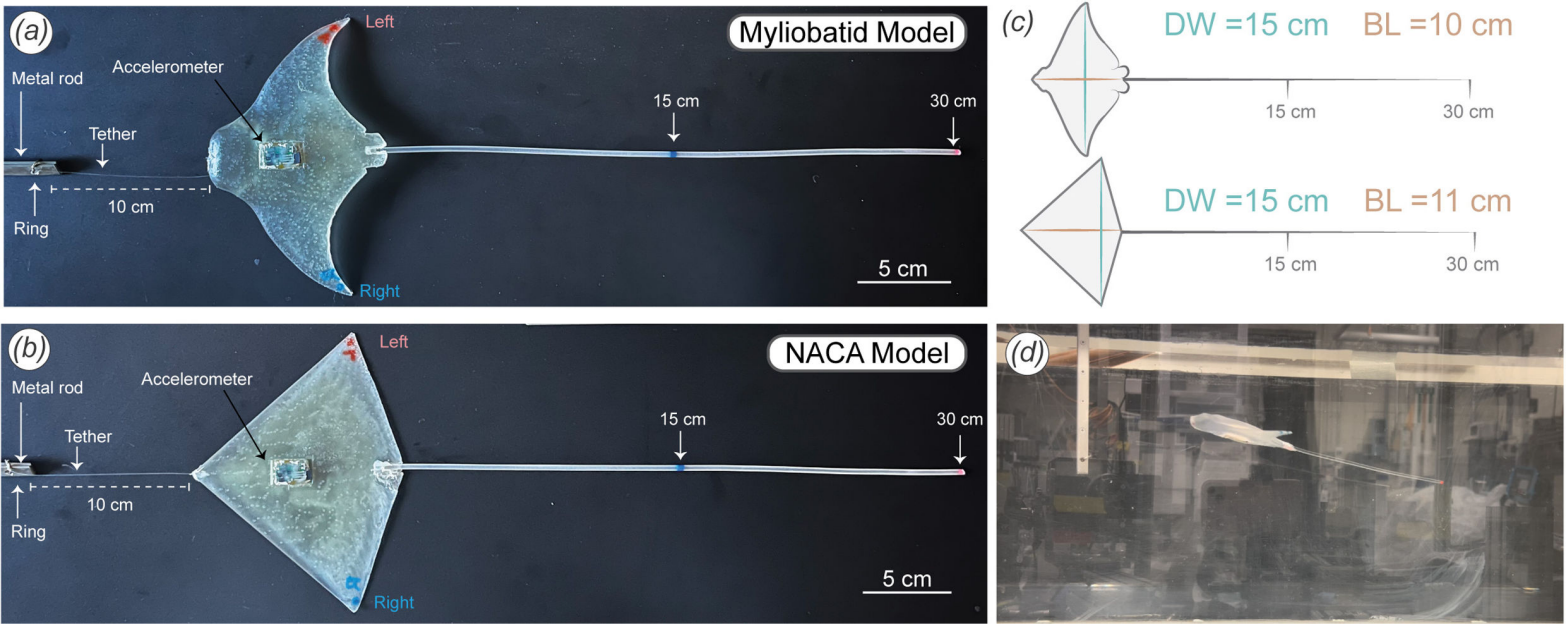
Models and experimental set-up. (*a*) Myliobatid model used in the experiments, with a tail marked at 30 cm (2 : 1 DW) and at 15 cm (1 : 1 DW). (*b*) NACA 0012 model with the tail marked at 30 cm (2 : 1 DW) and at 15 cm (1 : 1 DW). Both models were attached to a metal rod using an 8 pound fishing line 10 cm long attached to a ring and tied to the hole visible at the bottom of the vertical rod. The accelerometer was placed on the ventral side of the models. (*c*) Schematic representation of both models (DW and BL) and their respective tails. (*d*) Flow tank experimental set-up showing the myliobatid model with a 15 cm tail suspended in the recirculating flume with flow. The model has assumed a positive angle of attack and positive pitch at this speed.

We designed a myliobatid model using Maya 3D software 2024 (Autodesk Inc., San Francisco, CA, USA) (figure 2*a*) that represents a ‘generalized’ myliobatid body morphology. The model was based on three-dimensional models of myliobatids available online [45,46] but altered to ensure left-right symmetry by modelling one side of the body and mirroring it and adjusted several features (including body thickness, and the shape of the pectoral and pelvic fins) based on morphological data collected in this study, to make the model more representative of a real myliobatid. To determine the difference in mass between the body and the tail, we weighed the body and the tail of a *R. bonasus* specimen separately (specimen MCZ_49097 from the Harvard MCZ). For this specimen, the tail accounted for 1.3% of the total mass of the animal (body mass = 1543 g, tail mass = 21 g), and in our experiments, a tail of comparable relative length represented 2.3% of the total mass (body mass = 47.3 g, tail mass = 1.1 g). The myliobatid body model was designed with a DW of 15 cm and a BL of 10 cm (figure 2*a*,*c*).

Additionally, we generated an abstracted myliobatid model (using a NACA model aerofoil) with a simplified and known geometry using Maya 3D software 2024 (figure 2*b*). The ‘NACA model’ employed a NACA 0012 (National Advisory Committee for Aeronautics) cross-sectional profile in the streamwise dimension. We selected the NACA 0012 profile because it is symmetrical, maintains a high lift-to-drag ratio over a range of angles of attack, and has been previously used as a biomimetic shape to understand aquatic locomotion [38,47]. To generate a delta-like shape (similar to a myliobatid-like body shape), the chord length (and thus thickness) of the model decreased towards the wing tips, and the location of maximum wingspan matched that of the myliobatid model at approximately 77% of the BL. The NACA model was similar in weight (body mass = 43.7 g), where the tail represented 2.5% of the total mass, disc with a 15 cm DW and a BL of 11 cm (figure 2*b*,*c*).

We designed both three-dimensional-printed models with several internal cavities essential to experimental testing. On the ventral surface at the approximate centre of mass (which correlates with the position of the centre of mass measured in myliobatids [6]), we placed a rectangular cavity (2.3 × 1.3 × 0.9 cm) in the model to house an accelerometer datalogger. We added a small cavity (0.7 cm diameter) at the rostrum’s (anterior) midpoint for attaching a tether to secure the model during flow tank experiments. Finally, we created a small (0.3 cm diameter) cylindrical cavity along the posterior edge of the model between the pelvic fins to attach tails of varying length (figure 2*a*,*b*).

We three-dimensional-printed the models with the stereolithography printer FormLabs 3+ (FormLabs, Somerville, MA, USA). The resin used was *Elastic50A* V2, because its density (1.01 g ml^−1^) closely resembles that of measured myliobatid body densities (calculated above). Once printed, we inserted an accelerometer datalogger (Axy−5, TechnoSmart, Rome, Italy) into the ventral cavity of the model and fixed in place using hot glue (figure 2*a*,*b*). Prior to each trial, we attached a new tail of 30 cm in length to the cavity between the pelvic fins and fixed it in place using hot glue. We made the tails using a semitransparent, flexible thermoplastic polyurethane filament NinjaFlex (NinjaTek Inc., Manheim, PA, USA) with a diameter of 0.3 cm. We straightened the tails using heat with a heat gun to remove any residual curvature. We placed a blue mark at the 15 cm midpoint to mark half tail length, and a red mark at 30 cm to indicate the tail end (figure 2*a*,*b*).

### 2.3. Flow tank testing

We tested models with and without tails in a recirculating flow tank with varying flow speeds and tail lengths. The flow tank had a 28 × 28 × 66 cm (width, height, length) cross section and has been extensively used in previous work (figure 2*d*) [48,49]. To test the effects of the tail length on model stability, we implemented a towing protocol in which the physical models were attached from the rostral cavity to a rigid metal rod via a thin line (10 cm long, Sufix 8 pound monofilament line; Greensboro, NC, USA) (figure 2*a*,*b*,*d*, electronic supplementary material, videos S3–S5). The metal rod was vertically fixed in place so that the tether attachment was approximately 18 cm above the bottom of the tank, allowing the models to rest on the tank floor when there was no flow. This set-up ensured that the models maintained a streamwise orientation in the flow tank across a range of tested speeds, while allowing the acceleration data logger to measure body movements.

During the first set of experiments, we exposed both models (myliobatid and NACA) with a 30 cm tail (2× DW) to a flow speed of 7.5 cm s^−1^ (0.75 BL s^−1^) which was increased by 0.98 cm s^−1^ (or every 25 r.p.m. for the propeller) every 2 min in a stair step protocol, concluding at 46.8 cm^−1^ (4.6 BL s^−1^). We set the minimum speed at 7.5 cm s^−1^ because it was the lowest speed at which a model generated sufficient lift so that it was no longer resting on the bottom. After the maximum speed was reached, we cut the tail to 15 cm (1× DW) and repeated the speed sequence. Finally, we trimmed the tail to 0 cm (no tail) and repeated the experiments with the same speed sequence. We conducted this experimental protocol four times (i.e. four trials) for each model (NACA and the myliobatid). We recorded video data for all trials with a lateral-view camera.

To assess how small variations in tail length affected model stability, we conducted a second set of experiments in both the myliobatid and the NACA model. During the experiment, we gradually reduced the model’s tail length from 30 to 0 cm by cutting 1.5 cm from the tail every 2 min (e.g. 30, 28.5, 27, 25.5, etc.). We repeated this experiment three times for each model (i.e. three trials) at a constant speed of 14.1 cm s^−1^. We chose this speed as all models generated sufficient lift to raise the model to the mid-water level (figure 2*d*) and because of its biological relevance, falling within the known relative swimming speed range of myliobatid species [10]. Additionally, we also recorded high-speed camera footage from both lateral and bottom views of models in the flow tank at a speed of 1.4 BL s^−1^ to allow subsequent analysis of body motion (electronic supplementary material, videos S3–S5). Experimental Reynolds numbers ranged from approximately 7000 to 23 000 (based on a model length of 10 cm), which falls within the inertial flow regime, but is notably lower than the Reynolds numbers for large gliding myliobatid rays, which can be in the millions.

The use of a tether model to understand the hydrodynamic effect on stability on rays provides advantages as well as limitations. In hydrodynamic testing of models and robotic systems, several experimental and theoretical approaches are used. Some rely on simulations and theoretical models, others on experiments including rigid supports directly affixed to the model to measure force and motion [50–53], while others employ towing lines to simulate swimming and quantify drag [54–57]. The use of tethered models, despite their limitations, allows for controlled conditions difficult to replicate with live, free-swimming animals or small autonomous robotic platforms. Additionally, tethering allows simplification of experimental protocols, measurement of motion parameters during controlled testing conditions and repeatable, long-duration experiments. Although restricting motion of the anterior portion of the model with a tether attached at the nose reduces compensatory movements in pitch, this restriction is essential to isolate the passive hydrodynamic effect of the tail, which would otherwise be masked by active corrections or other hydrodynamic variables in free-swimming systems. The nose tether also limited the range of yaw and sway (side to side) movements, although side and lateral rotational movements are included in the ODBA measurements described below.

### 2.4. Data analysis

During all experiments, the embedded acceleration datalogger recorded pressure and temperature at 1 Hz, and tri-axial acceleration at 100 Hz. At the conclusion of each experimental trial, we downloaded the datalogger data. We aligned the dataloggers within the models so that the accelerometers’ *x*-axis corresponded to the rostral–caudal axis, the *y*-axis corresponded to the medial–lateral axis, and the *z*-axis corresponded to the dorsal–ventral axis of the models. The datalogger records proper acceleration, or the acceleration relative to free-fall, which can be decomposed into two components, static and dynamic acceleration. Static acceleration is acceleration due to gravity (9.8 m s^−2^) and as this vector is fixed in the global frame it can be used to reconstruct the posture (pitch and roll) of the model. Body pitch is the angle between the horizontal and the model’s rostral–caudal axis (i.e. rotation around *y*-axis), while body roll is the angle between the model’s medial–lateral axis and the horizon (i.e. rotation around the *x*-axis). We calculated pitch and roll of the models at each moment in time using equations (2.2) and (2.3)


(2.2) $$Roll={tan}^{1}(Y_{accel},Z_{accel}),$$

(2.3) $$Pitch=\tan^{-1}\left(-X_{accel},Y_{accel}\;\sin_{Roll}+Z_{accel}\;\cos_{Roll}\right).$$

A positive pitch angle indicates that the front of the model is tilted upwards, while a negative pitch angle indicates that the model is tilted downwards (figure 1*b*). A positive roll value corresponds with a model tilting to the right, while a negative roll value corresponds to the value tilting to the left (figure 1*b*). However, the recorded acceleration values additionally contain dynamic acceleration, or the acceleration due to changes in speed and orientation of the model. We calculated static acceleration using a 3 s running mean (300 point) of the raw acceleration data. We calculated dynamic acceleration as the remainder when subtracting the static acceleration from the raw acceleration [58]. From dynamic acceleration, we calculated ODBA (equation (2.4)), a metric that captures the degree of three-dimensional movement of an object and has been extensively used to capture animal activity levels [59].


(2.4) $$ODBA=abs(X_{dyn})+abs(Y_{dyn})+abs(Z_{dyn}).$$

In this study, we did not measure the third Euler angle defining body orientation, yaw, the orientation in the horizontal plane. An accelerometer cannot provide information on yaw, as it operates around the gravity vector, which is used to determine pitch and roll. Measuring yaw from a tag accurately requires additional sensors such as a magnetometer and gyroscope. While technically the tag employed in these studies included a magnetometer, it only recorded at 2 Hz, which aliased the yaw movements and magnetometer-only-derived headings can be inaccurate [60]. However, trends in yaw should similarly track orientation estimates of pitch and roll, as any change in orientation changes the force balance acting on the model. Additionally, while not explicitly measured, the rotational pivoting movements (yawing) around the attachment point of the tether at the nose of the model are accelerations that are recorded and thus incorporated in ODBA.

In aeronautics, stability is often described using two separate concepts: dynamic and static stability. Static stability refers to an object’s initial tendency to respond after a disturbance. An object with positive static stability will counter a disturbance, returning towards its original orientation, and negative static stability would be an amplification of the disturbance. As an object tends to return to its initial orientation, it often will overshoot, inducing an oscillation. Static stability captures this temporal trend induced from a disturbance, which is described as the object’s dynamic stability. An object that is positively dynamically stable will return to its initial orientation rapidly, with little oscillation, while an object with negative dynamic stability will continuously overshoot its initial orientation, inducing amplifying oscillations. In classical studies of stability, an untethered object is disturbed and the forces acting on it can be used to determine its static stability, while dynamic stability is assessed as how the object’s motion evolves over time. A common example is the arrow: when launched, the arrow flexes and undergoes small oscillations that gradually decrease until it straightens out, with pitch, yaw and roll stabilized by the feathers.

In this study, our models are tethered and not explicitly disturbed, limiting our ability to directly measure static or dynamic stability. However, each speed change and the small deviations from laminar flow can be interpreted as constant sources of disturbances for our models. Thus, we assessed stability as the degree of movement of the model during each experiment. Models that display high degrees of movement would have negative dynamic and/or static stability, while models that only display small variations in movement would have positive dynamic and static stability.

For statistical analysis, we collapsed the tag data into 2 min bins based on speed and tail length for each trial. We removed the first 20 s of each treatment after manipulation (i.e. speed change or tail cutting) so that only the steady-state behaviour was captured. From each trial bin (speed, tail and model type), we summarized the mean body pitch and body roll to understand the orientation and position of the model and quantified the degree of movement and thus stability of the model using three metrics: the mean ODBA, the range (maximum–minimum) of the model’s roll and pitch. The pitch and roll range capture the largest orientation space that the model occupies. Models with larger pitch and roll ranges would experience larger amplitude orientation ranges and thus decreased stability. The mean ODBA, captures both linear accelerations as well as higher frequency (greater than 0.33 Hz) changes in orientation, and thus is able to capture the degree of movement of the model consistently through the trial. This metric allows for the movement to be captured, regardless of the direction or movement or orientation change and has been used extensively to describe behavioural activity levels in free swimming animals [59,61]. Models with lower mean ODBAs will experience decreased movement and thus increased stability.

### 2.5. Statistical analysis

To examine morphological variation across myliobatid species, we analysed a series of linear models, and to examine the relationship between tail length and body size, we conducted regressions of tail length against BL. Species with less than three individuals were not included in the statistical analysis, except for *Mo. birostris*. Despite being represented by only two individuals, *Mo. birostris* was included in the statistical analysis because it is the largest myliobatid species and morphological data on this species are rare. In these models, we incorporated an interaction between body size (continuous variable) and species (discrete factor), allowing each species to have their own slope and intercept. To test if the tail length varied with the body shape among myliobatid species, we analysed the standardized tail length (tail length/BL) against the AR (figure 1) for each myliobatid species with ANOVA. To determine which species contained significantly different standardized tail length relative to body shapes (standard tail length, AR), we conducted post hoc comparisons using the Tukey honestly significant difference (HSD) test.

To analyse how flow speed and tail length impacted body position (pitch, roll) and stability (ODBA, pitch range, roll range) metrics, we constructed linear models. Body position and stability metrics were response variables, while tail length, flow speed and the interaction between tail length and flow speed were the predictor (independent) variables. Tail length and flow speed were classified as discrete factors and not continuous variables. Post hoc analysis included pairwise comparisons (*t*‐test) using planned contrasts (*n* = 24), so that only comparisons between tail lengths at the same speed were included. We adjusted the significance value of post hoc tests to account for multiple testing using the Bonferroni correction. We constructed the statistical models separately for the myliobatid and NACA models.

To determine if there were significant differences between the NACA and myliobatid models, we constructed a separate series of statistical models. First, we conducted linear models with positional and stability metrics as response variables, while the predictor (independent) variables were model type (myliobatid, NACA), flow speed and the interaction between them. Then, we conducted separate models for each tail length (30, 15 and 0 cm). We used *t*-tests as post hoc pairwise comparisons so that only models were compared with the same tail lengths and at the same speed.

Models with 30 and 15 cm tails had their tail in contact with the bottom of the tank at the lowest tested speed. As this altered their movement, all 7.5 cm s^−1^ speed measurements were excluded from analysis. In order to facilitate comparison across studies, we have presented speeds in units of BLs per second, BL s^−1^, by dividing the flow speed by the model’s BL. All data and statistical analyses were conducted in R (R Foundation for Statistical Computing, Vienna, Austria, version 4.4.2).

## 3. Results

### 3.1. Myliobatid morphology

**Figure 3.**
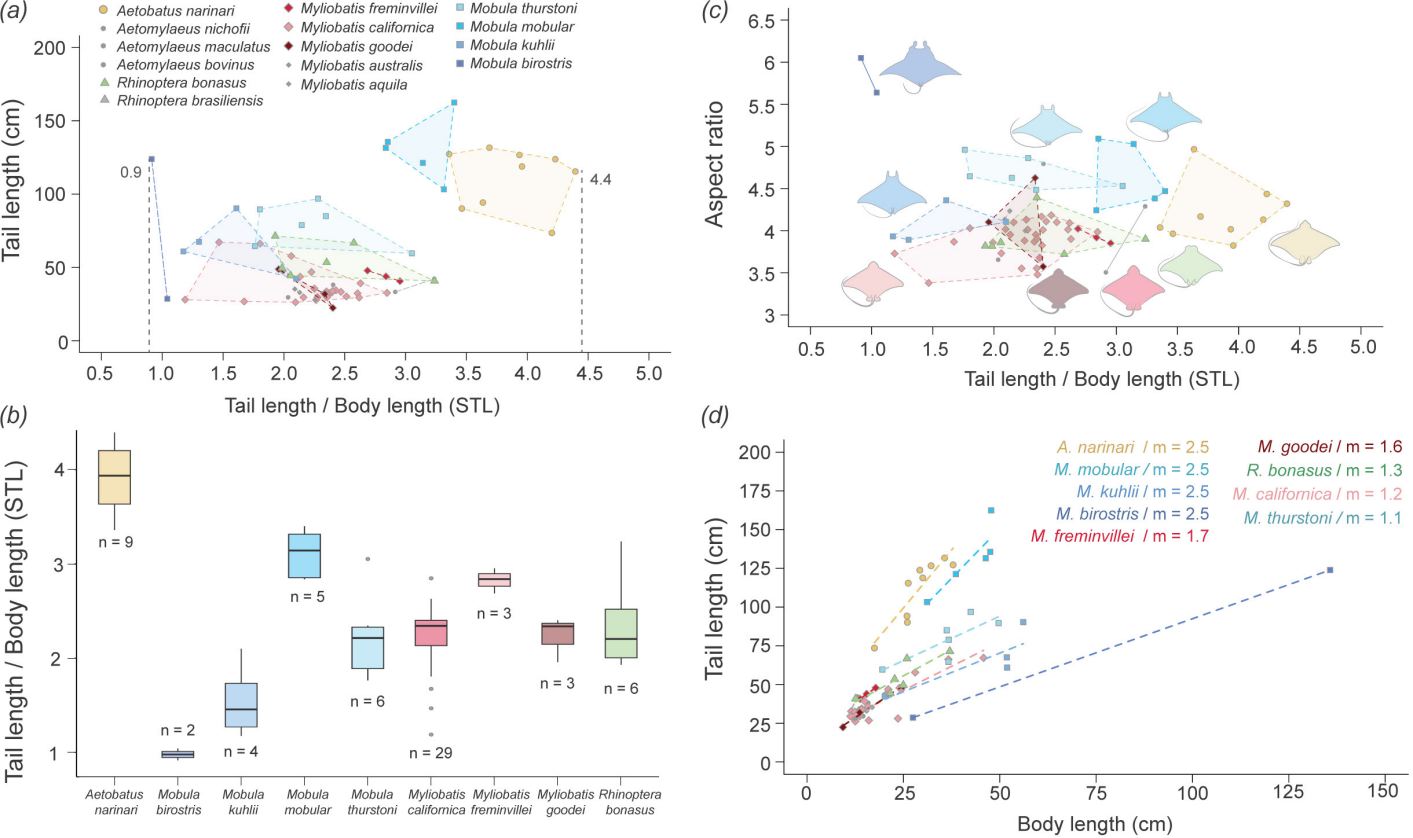
Tail length variation across myliobatid species. (*a*,*b*) Variation in standardized tail length (STL; tail length/BL) among myliobatid species (n = 76). Only species with more than two individuals were statistically analysed, except for *Mo. birostris* (*n* = 2). Statistically analysed species are highlighted in different colours, while non-analysed species (*n* < 3) are shown in grey. STL varied across different myliobatid species (ANOVA, *p* < 0.001), with *Mo. birostris* having the shortest STL (0.9 BL) and *Aetobatus narinari* the longest (4.4 BL). *Mobula thurstoni* exhibited the longest absolute tail length recorded. (*c*) Relationship between STL and AR across myliobatid species. Mobulids consistently showed the highest AR compared with other myliobatids. *Mobula birostris* had the highest AR and *My. californica* had the lowest AR. In contrast, *A. narinari* displayed a low AR but the longest tail. (*d*) Correlation between tail length and BL for species with more than two specimens measured. For all species, tail length increased with BL (ANOVA, *p* < 0.001) but at different rates among species (ANOVA, *p* < 0.001). *Aetobatus narinari*, followed by *Mo. mobular*, the tail length increased at higher rate relative to BL (higher *m* values), contrasting with *Mo. birostris*, whose tail length increased at the lowest rate relative to BL.

We analysed how tail length varied with BL, DW and AR across 15 different species (*n* = 76 individuals) from the four oscillatory families of Myliobatiformes (Mobulidae, Rhinopteridae, Myliobatidae and Aetobatidae). In this study, we refer to these four families collectively as myliobatids. The specimens available were mainly juveniles and presented a large variation in tail length (TL, 22−162 cm), BL (9−136 cm) and DW (16−338 cm, figure 3). Mobulids were the largest myliobatids, with *Mo. mobular* (giant devil ray) possessing the longest tail (TL = 162 cm) and *Mo. birostris* (giant manta ray) the largest body size (BL = 136 cm, DW = 338 cm) (figure 3). Across all measured species, tail length increased with BL (*F*_1,49_ = 671, *p* < 0.001) and DW (*F*_1,49_ = 558, *p* < 0.001). However, the rate at which the tail increased varied among myliobatid species (BL: *F*_8,49_ = 4.3, *p* < 0.001; DW: *F*_8,49_ = 5.5, *p* < 0.001) (figure 3*d*).

To calculate the standardized tail length (STL) across myliobatid species, we calculated the tail-to-BL ratio (tail length/BL). Our results showed that the STL significantly differed among species (ANOVA, *F*_8,58_ = 28, *p* < 0.001), ranging from 0.9 in *Mo. birostris* (meaning that the tail is 0.9 times the BL) to 4.4 in *Aetobatus narinari* (spotted eagle ray, where the tail is 4.2 times the BL) (figure 3*a*,*b*). In fact, *A. narinari* had significantly longer tails (STL = 3.8 ± 0.3 BL) than any other species (Tukey HSD, *p* < 0.01). While *Mo. birostris* only had two observations precluding robust statistical analysis, these were the two shortest tails observed in this study (STL = 0.9 ± 0.1 BL) (figure 3*a*,*b*). For species with more than five observations, the range between the shortest and longest tail within a species always exceeded 1 BL (figure 3). Regarding the STL for other species, myliobatids from the families Rhinopteridae (*R. bonasus*_STL_ *=* 2.4 ± 0.5 BL)*,* Myliobatidae (*My. californica*_STL_ = 2.2 ± 0.4 BL) and Mobulidae (*Mo. thurstoni*_STL_ = 2.2 ± 0.5 BL) displayed overlapping tail lengths that did not differ from each other (Tukey HSD, *p* > 0.9). Notably there was variation within mobulids, with *Mo. mobular* (STL = 3.1 ± 0.3 BL) having significantly (Tukey HSD, *p* = 0.003) longer tails than *Mo. thurstoni* (STL = 2.2 ± 0.5) and *Mo. kuhlii* (STL = 1.5 ± 0.4). With the exception of *Mo. birostris* (which was included in the analysis due to its large size), species with less than three individuals were not statistically analysed but plotted in the graphs (grey points in figure 3*a*,*c*). These species included *A. nichofii, A. maculatus, A. bovinus, R. brasiliensis, My. australis* and *My. aquila*. These species presented similar STL values to other individuals of their corresponding families.

Similar to STL, there was significant variation in AR across species (ANOVA, *F*_8,58_ = 19, *p* < 0.001) (figure 3*c*). *Mobula birostris* had the highest AR (5.8 ± 0.3) and two other species of *Mobula (Mo. thurstoni*_AR_ = 4.7 ± 0.2; *Mo. mobular*_AR_ = 4.6 ± 0.4) presented a higher AR than other myliobatids (Tukey HSD, *p* < 0.1). However, the AR of *Mo. kuhlii* (AR = 4.1 ± 0.2), *A. narinari* (AR = 4.2 ± 0.3), *R. bonasu*s (AR = 3.9 ± 0.2) and *Myliobatis* sp. (*My. freminvillei*_AR_ = 3.9 ± 0.1; *My. californica*_AR_ = 3.9 ± 0.2), did not differ from each other (Tukey HSD, *p* > 0.9) (figure 3*c*).

By measuring the weight in air and in ethanol, we were able to estimate the density of 10 myliobatid species (*n* = 34 individuals). There was no significant difference in density among species (*F*_10,23_ = 0.9, *p* = 0.51) or with BL (*F*_1,28_ = 2.5, *p* = 0.11). However, we were only able to measure individuals less than 33.7 cm DW. These measurements provided an approximate reference for the models tested in the flow tank.

### 3.2. Model posture

**Figure 4.**
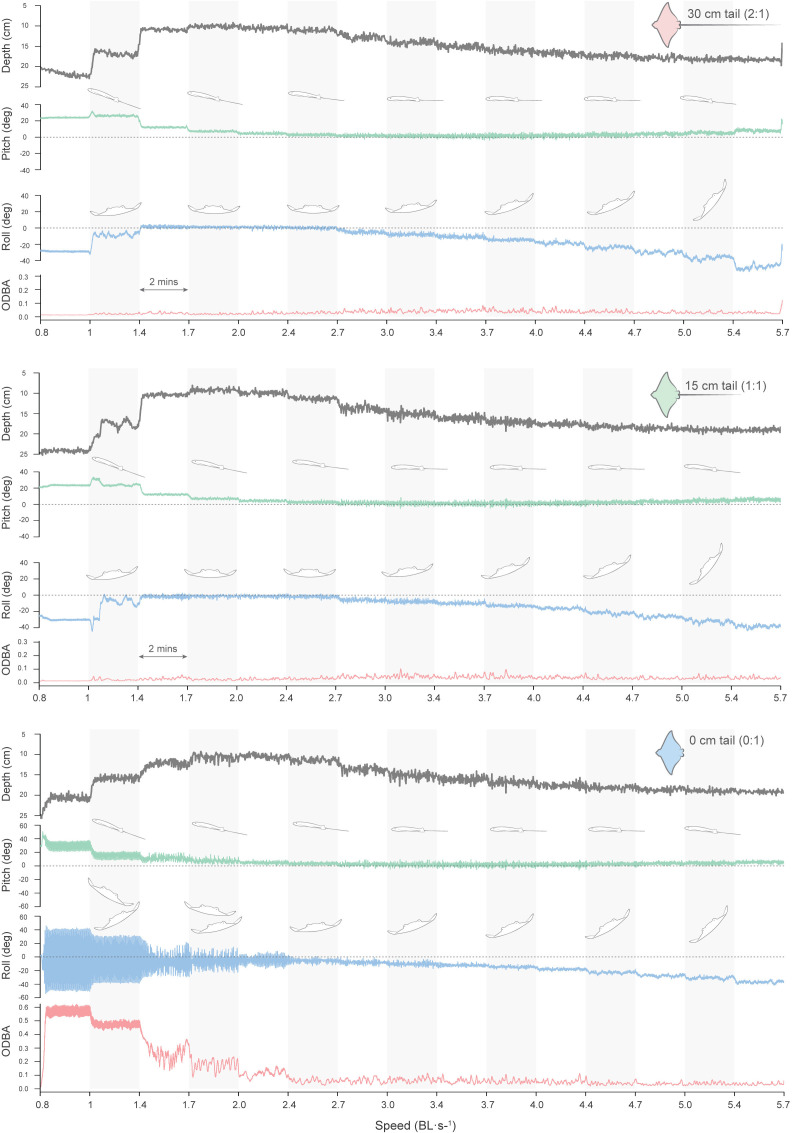
Depth, pitch, roll and ODBA variation of the myliobatid model across increasing flow speed and different tail lengths in a single trial. Horizontal bars (grey and white) indicate flow speed intervals, each maintained for 2 min. The myliobatid model without a tail exhibited a significant increase in pitch, roll and ODBA. Notably, the model tended to tilt to the right as flow speed increased.

**Figure 5.**
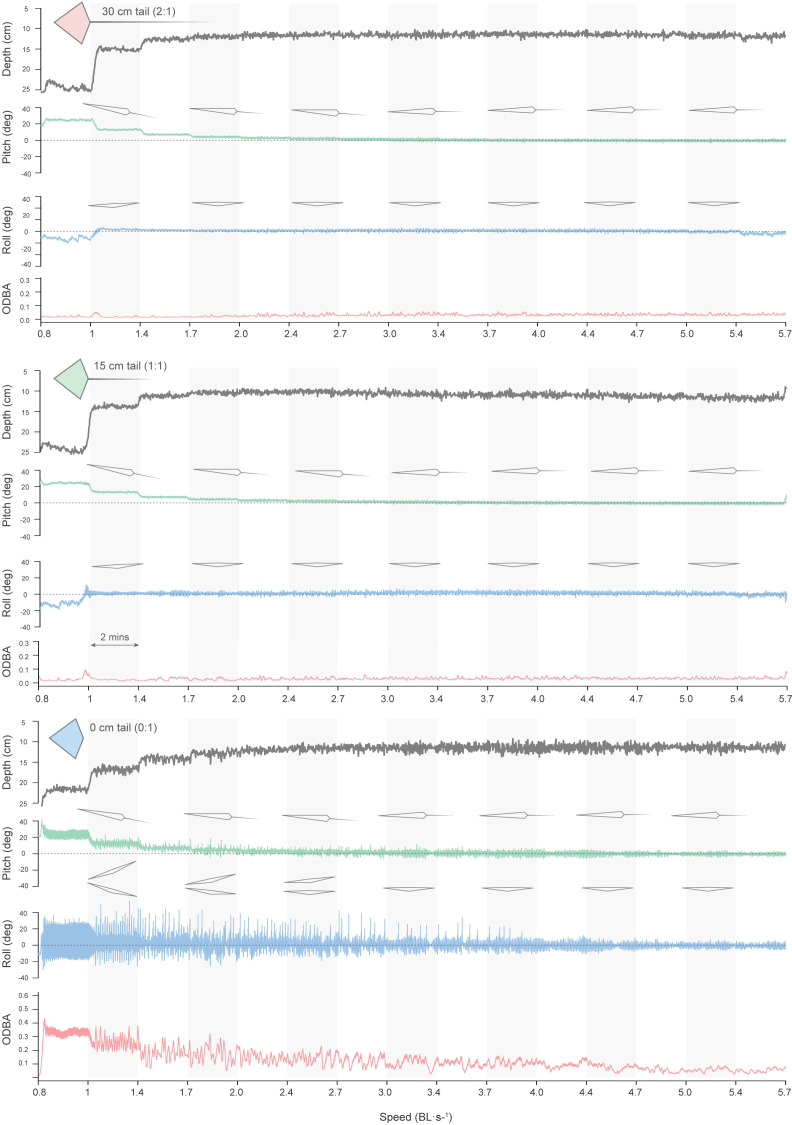
Depth, pitch, roll and ODBA variation of the NACA 0012 model across increasing flow speeds and different tail lengths in a single trial. Horizontal bars (grey and white) indicate flow speed intervals, each maintained for 2 min. The NACA model without a tail had a higher pitch, roll and ODBA that decreased with higher flow speeds than the myliobatid model. Note that this model does not tilt to one side as the speed increases.

**Figure 6.**
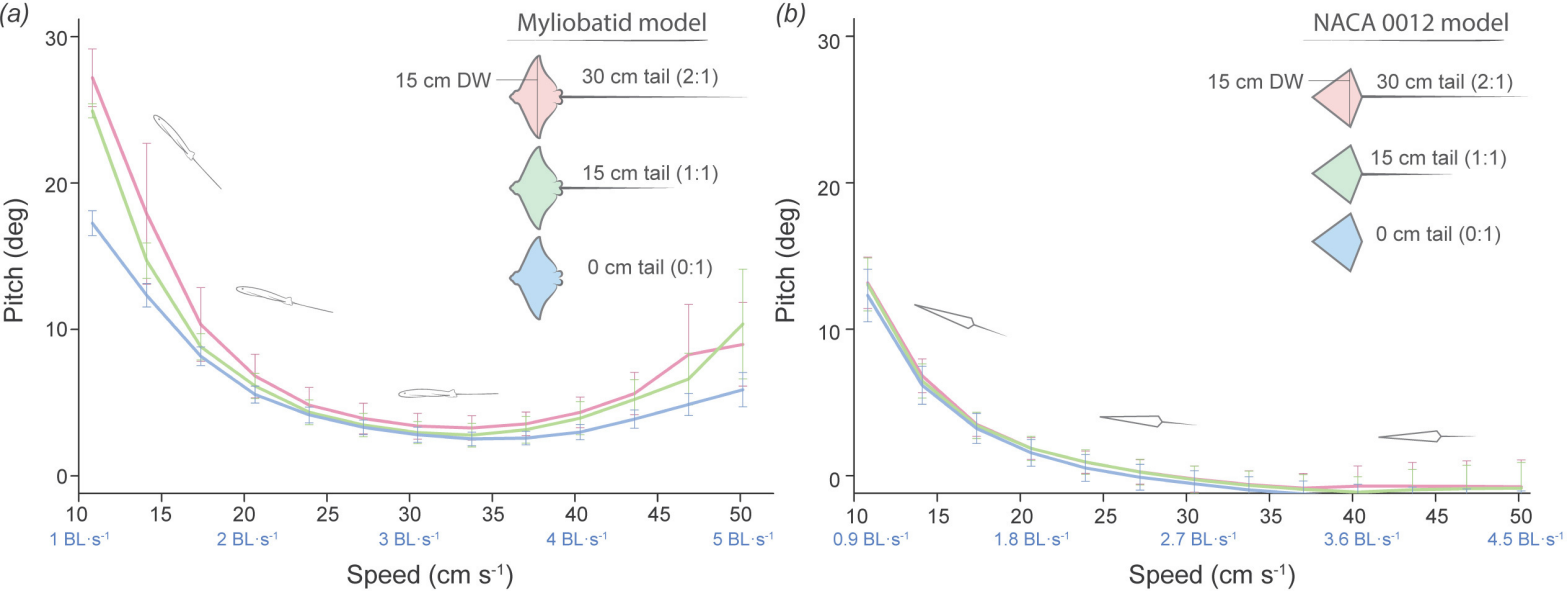
Variation of mean pitch values across flow speeds and tail lengths. Lines of different colours on the graph represent each tail length (30, 15, 0 cm). Error bars indicate the standard error based on four repeated trials for each tail length. Flow speeds are indicated as cm s^−1^ and BL s^−1^ (BL). Overall**,** both models presented their maximum pitch value at lower flow speeds and, as the flow speed increased, the pitch significantly decreased (ANOVA, *p* < 0.001). (*a*) For the myliobatid model, both tail lengths (30 and 15 cm) produced higher mean pitch values than the model without a tail (Tukey HSD, 0:15 cm: *p* < 0.001; 0:30 cm: *p* = 0.005). However, as flow speed increased, no significant differences were observed between different tail lengths (Tukey HSD, *p* > 0.1). (*b*) The NACA 0012 model presented no differences in pitch average among all tail lengths (ANOVA, *p* = 0.38) and displayed a lower pitch angle than the myliobatid model (ANOVA, *p* < 0.01).

**Figure 7.**
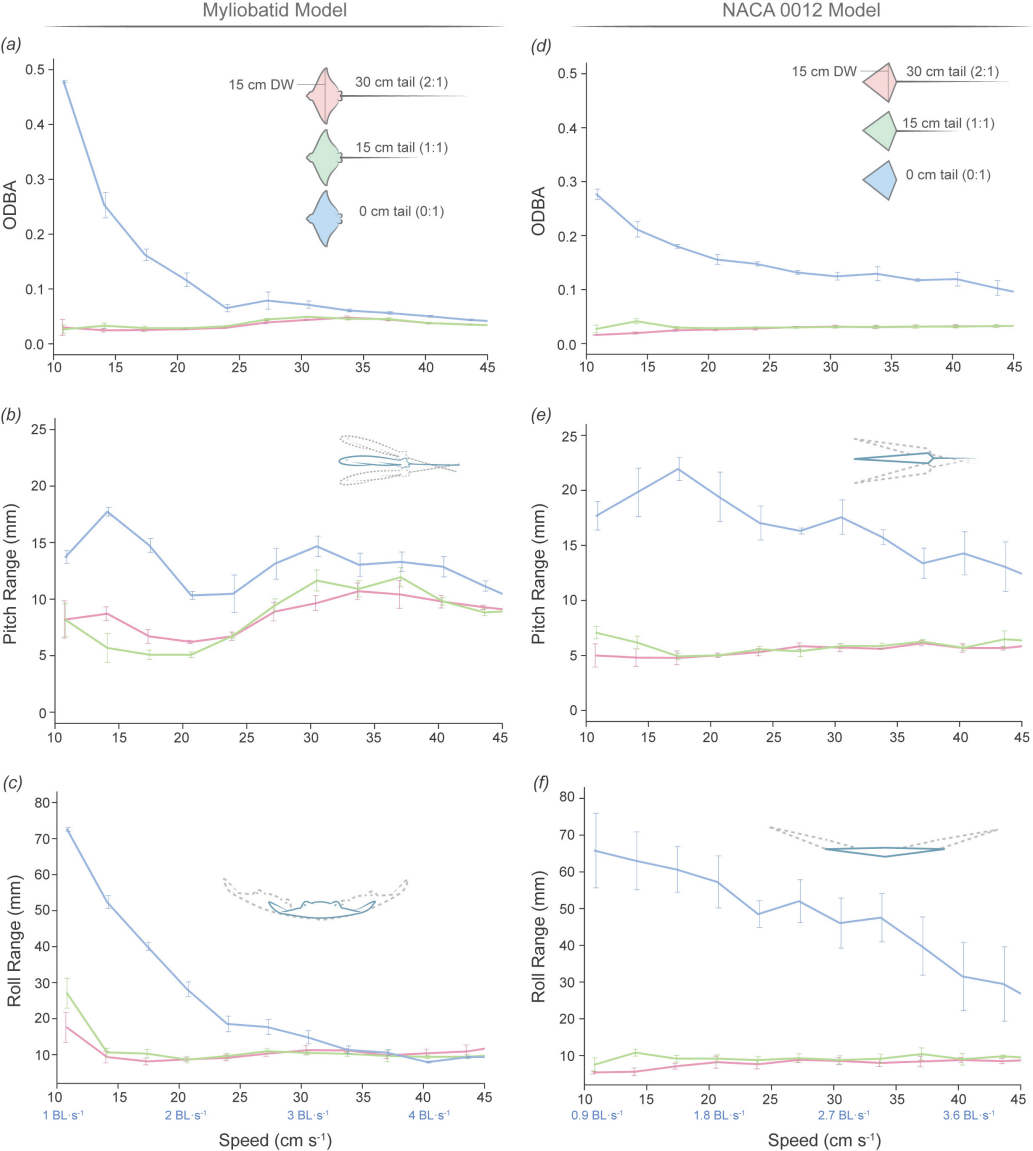
Variation in pitch range, roll range and ODBA for both models across tail lengths and flow speeds. Lines of different colours on the graph represent each tail length (30, 15, 0 cm). Error bars indicate the s.e. based on four repeated trials for each tail length. Flow speeds are indicated as cm s^−1^ and BL s^−1^ (BL). (*a*) ODBA variations in the myliobatid model. Models with tails maintained consistent ODBA across all speeds, without significant differences among both tail lengths (post hoc *t*‐test, *p* > 0.05), whereas models without tails exhibited significantly higher ODBA, particularly at speeds below 2.5 BL s^−1^ (25 cm s^−1^) (post hoc *t*‐test, *p* < 0.05). (*b*) Pitch range variations in the myliobatid model. Models without tails showed a greater pitch range than those with tails across all speeds (post hoc *t*‐test, *p* < 0.05). (*c*) Roll range variations in the myliobatid model. At speeds below 3 BL s^−1^ (30 cm s^−1^), models without tails exhibited a higher roll range than those with tails (post hoc *t*‐test, *p* < 0.05). (*d*) ODBA variations in the NACA 0012 model. NACA models without tails exhibited higher ODBA than those with tails at all speeds (post hoc *t*‐test, *p* < 0.05). (*e*) Pitch range variations in the NACA model. Models without tails significantly increased the pitch range at all speeds (post hoc *t*‐test, *p* < 0.05). (*f*) Roll range variations in the NACA model. Models without tails presented larger roll ranges at all speeds; being particularly high at lower speeds and decreasing as speed increased (post hoc *t*‐test, *p* < 0.05).

At the lowest speed (10 cm s^−1^ or 1 BL s^−1^), both the NACA and myliobatid models displayed their maximum pitch value (tilted up posture, with a positive body angle of attack to oncoming flow) (figures 4–6, table 1; electronic supplementary material, table S3). As flow speed increased, the pitch of the models significantly decreased (myliobatid: *F*_15,134_ = 78, *p* < 0.01, NACA: *F*_15,132_ = 99, *p* < 0.01) (figures 4–6, table 1; electronic supplementary material, table S3) and models were approximately horizontal (pitch < 5°) once speeds reached 3 BL s^−1^ (30.5 cm s^−1^) (figures 4–6, table 1; electronic supplementary material, table S3). While the NACA model’s roll (roll < 3°; electronic supplementary material, table S3) did not change with flow speed (*F*_15,132_ = 0.52, *p* = 0.92) (figure 5), once speed exceeded 3.0 BL s^−1^ the myliobatid model displayed a progressive roll to the right-hand side (*F*_15, 132_ = 18.6, *p* < 0.001) (figure 4; table 1). As the speed increased, the myliobatid model’s roll could exceed 45° and it would turn upside down (roll = 180°) at speeds faster than 5.7 BL s^−1^ (57.7 cm s^−1^) (figure 4). As the myliobatid model’s roll increased, the estimated pitch of the model also displayed minor increases (pitch < 5°) (figures 4 and 7*b*, table 1). Due to this rolling behaviour, speeds higher than 4.7 BL s^−1^ (47 cm s^−1^) were not included in the statistical tests for any of the models.

**Table 1.** Mean ± s.e. values for pitch, pitch range, roll, roll range and mean ODBA across speeds for the myliobatid model.

speed cm s^−1^	speed BL s^−1^	pitch mean | pitch range (°)						roll mean | roll range (°)						ODBA (g)		
		30 cm		15 cm		0 cm		30 cm		15 cm		0 cm		30 cm	15 cm	0 cm
10.8	1.1	27.2 ± 1.7	8.2 ± 1.4	24.9 ± 0.4	8.1 ± 1.2	17.2 ± 0.7	13.7 ± 0.4	2.1 ± 9.3	17.5 ± 3.6	−8.5 ± 1.4	27.1 ± 3.5	−4.1 ± 0.4	72.6 ± 0.4	0.02 ± 0.01	0.03 ± 0.006	0.48 ± 0.001
14.1	1.4	17.9 ± 4.1	8.7 ± 0.5	14.7 ± 1.0	5.6 ± 1.1	12.3 ± 0.6	17.7 ± 0.3	−1.5 ± 1.8	9.3 ± 1.4	−3.5 ± 0.8	10.6 ± 1.0	−6.9 ± 1.5	52.4 ± 1.5	0.02 ± 0.002	0.03 ± 0.004	0.25 ± 0.02
17.3	1.7	10.3 ± 2.1	6.7 ± 0.5	8.8 ± 0.7	5.1 ± 0.3	8.1 ± 0.5	14.7 ± 0.5	−3.2 ± 1.5	8.1 ± 0.8	−3.5 ± 0.7	10.3 ± 1.0	−5.7 ± 0.9	40.1 ± 0.9	0.02 ± 0.002	0.03 ± 0.002	0.16 ± 0.009
20.6	2.1	6.8 ± 1.2	6.2 ± 0.1	6.1 ± 0.7	5.1 ± 0.2	5.5 ± 0.4	10.3 ± 0.2	−3.7 ± 1.6	8.7 ± 0.3	−3.9 ± 0.9	8.6 ± 0.7	−4.8 ± 1.7	28.1 ± 1.7	0.03 ± 0.0004	0.03 ± 0.001	0.12 ± 0.01
24.0	2.4	4.8 ± 1.0	6.7 ± 0.3	4.3 ± 0.7	6.8 ± 0.1	4.2 ± 0.4	10.4 ± 1.4	−4.9 ± 1.6	9.1 ± 0.7	−5.3 ± 1.1	9.6 ± 0.6	−5.5 ± 1.8	18.5 ± 1.8	0.03 ± 0.0009	0.03 ± 0.001	0.06 ± 0.005
27.2	2.7	3.9 ± 0.9	8.9 ± 0.7	3.5 ± 0.6	9.5 ± 0.4	3.3 ± 0.4	13.1 ± 1.1	−8.7 ± 1.2	10.3 ± 0.6	−8.8 ± 0.8	10.9 ± 0.5	−7.7 ± 1.8	17.7 ± 1.8	0.04 ± 0.002	0.04 ± 0.001	0.08 ± 0.01
30.5	3.1	3.3 ± 0.7	9.6 ± 0.5	2.9 ± 0.6	11.6 ± 0.8	2.8 ± 0.4	14.6 ± 0.7	−11.7 ± 1.4	11.3 ± 1.1	−11.6 ± 1.2	10.5 ± 0.4	−10.0 ± 1.5	14.8 ± 1.5	0.04 ± 0.001	0.05 ± 0.001	0.07 ± 0.005
33.8	3.4	3.2 ± 0.7	10.7 ± 0.6	2.8 ± 0.6	10.9 ± 0.6	2.5 ± 0.4	13.1 ± 0.8	−14.9 ± 1.6	11.2 ± 0.4	−14.4 ± 1.4	10.2 ± 0.5	−12.2 ± 0.9	11.3 ± 0.9	0.05 ± 0.002	0.04 ± 0.0001	0.06 ± 0.01
37.0	3.7	3.5 ± 0.6	10.4 ± 1.0	3.1 ± 0.7	11.9 ± 0.7	2.6 ± 0.4	13.3 ± 0.7	−18.9 ± 1.9	9.8 ± 0.8	−18.3 ± 1.8	9.6 ± 0.4	−14.8 ± 0.6	10.5 ± 0.6	0.04 ± 0.002	0.04 ± 0.001	0.06 ± 0.005
40.3	4.0	4.3 ± 0.9	9.7 ± 0.4	3.9 ± 0.9	9.8 ± 0.2	2.6 ± 0.4	12.8 ± 0.7	−24.0 ± 2.6	10.4 ± 0.9	−23.1 ± 2.4	9.4 ± 1.4	−18.5 ± 0.2	7.9 ± 0.2	0.04 ± 0.002	0.04 ± 0.0001	0.05 ± 0.002
43.6	4.3	5.6 ± 1.2	9.2 ± 0.1	5.2 ± 1.1	8.8 ± 0.2	2.9 ± 0.5	11.1 ± 0.7	−30.2 ± 3.3	10.9 ± 1.5	−29.5 ± 2.7	9.4 ± 0.4	−23.3 ± 0.2	9.3 ± 0.2	0.03 ± 0.001	0.03 ± 0.001	0.04 ± 0.002
46.9	4.6	8.2 ± 2.9	8.8 ± 0.3	6.6 ± 1.5	9.0 ± 0.2	3.9 ± 0.6	9.6 ± 0.3	−39.9 ± 7.6	12.7 ± 2.5	−35.6 ± 3.8	10.1 ± 0.4	−28.1 ± 0.1	9.3 ± 0.1	0.03 ± 0.0009	0.03 ± 0.002	0.04 ± 0.001

Tail length (0, 15, 30 cm) had only minor effects on the posture of the models (pitch, roll) during testing (figure 6). In general, tail length did not significantly affect either model’s roll (myliobatid: *F*_2,134_ = 0.966, *p* = 0.38; NACA: *F*_2,132_ = 1.02, *p* = 0.36). For the NACA model, tail length did not significantly affect average pitch (*F*_2,132_ = 1.28, *p* = 0.28) (figure 5, electronic supplementary material, table S3), regardless of speed (*F*_32,132_ = 0.13, *p* > 0.99) (figure 6*b*). In contrast, for the myliobatid model, tail length significantly impacted body pitch (*F*_2,134_ = 4.9, *p* = 0.009), although this effect was not uniform across speeds (*F*_28,134_ = 2.12, *p* = 0.002) (figure 6*a*). At the lowest speeds (1 BL s^−1^), myliobatid models with tails had higher mean pitch values (30 cm tail = 27.20 ± 1.7; 15 cm tail = 24.93 ± 0.4) than the model without a tail (0 cm tail = 17.25 ± 0.7) (Tukey HSD, 0 : 15 cm: *p* < 0.001; 0 : 30 cm: *p* = 0.005) (table 1; figure 6*a*). However, as flow speed increased, no significant differences in body pitch were observed between myliobatid models with different tail lengths (Tukey HSD, *p* > 0.1) (figure 6*a*).

There were differences in the average posture of the NACA and myliobatid models. The myliobatid model displayed higher pitch values than the NACA model (0 cm: *F*_1,78_ = 261, *p* < 0.001; 15 cm: *F*_1,78_ = 172, *p* < 0.001; 30 cm: *F*_1,78_ = 119, *p* < 0.001), and this was true at all speeds (10–50 cm s^−1^, pairwise *t*‐test *p* < 0.05) (table 1, electronic supplementary material, table S3). As the myliobatid models rolled onto their sides at increasing speed (i.e. tilted to the right) and the NACA models did not, there were significant differences in the roll posture between the models (0 cm: *F*_1,78_ = 3329, *p* < 0.001; 15 cm: *F*_1,78_ = 279, *p* < 0.001; 30 cm: *F*_1,78_ = 148, *p* < 0.001). However, at low speeds (less than 225 r.p.m.), there were no differences between the average roll position of the NACA and myliobatid models (paired *t*‐test, *p* > 0.05).

### 3.3. Model stability

To quantify model stability, we used three variables that described the movement of the model: (i) the ODBA combines the dynamic acceleration of an animal’s body across *X*, *Y* and *Z* axes; (ii) pitch range, that quantifies the movement of the model in *Y–Z* (anterior–posterior) plane; and (iii) roll range, that quantifies the movement of the model in the *X*–*Y* (medial–lateral) plane. Flow speed and tail length had a large impact on all three variables, and therefore, an effect on the model stability (figure 7; electronic supplementary material, tables S4–S6).

Regardless of flow speed, both the myliobatid and NACA models without tails (0 cm) moved significantly more (higher ODBA) than either of the models with tails (15 and 30 cm; post hoc *t*‐test *p* < 0.05) (figure 7*a*,*d*; table 1; electronic supplementary material, tables S4–S6). These stability differences between tailed and tailless models were most pronounced at the lowest flow speed (1.0 BL s^−1^) in which the tailless models had an order of magnitude higher ODBA (myliobatid: 0 cm tail = 0.48 ± 0.001 g; NACA model: 0 cm = 0.28 ± 0.008 g) than the tailed models (ODBA < 0.03 g) (table 1 and electronic supplementary material, table S1). As flow speed increased, the ODBA of the tailless NACA and myliobatid models decreased (indicating decreased movement) (figure 7*a*,*d*). However, as the flow speed increased (speed > 3 BL s^−1^), the myliobatid model approached ODBA values similar to myliobatid models with tails (figure 7*a* and table 1), the tailless NACA model maintained a higher ODBA than those with tails for all speeds (figure 7*d* and electronic supplementary material, table S1).

Doubling the tail length from 15 to 30 cm, did not significantly change the ODBA of either the NACA or myliobatid models in over 90% of tested speeds (post hoc *t*‐test *p* > 0.05) (electronic supplementary material, tables S4–S6). Additionally, while there were significant differences between the myliobatid and NACA model’s ODBA (0 cm: *F*_1,78_ = 53, *p* < 0.001; 15 cm: *F*_1,78_ = 23, *p* < 0.001; 30 cm: *F*_1,78_ = 24, *p* < 0.001), at speeds below 2.4 BL s^−1^ the NACA and myliobatid models with 15 and 30 cm tails did not differ from each other (post hoc *t*‐test: *p* > 0.05).

To quantify how the models were moving, we further assessed the variability in model posture at each speed using pitch range and roll range variables. Similar to ODBA, models with tails (15 and 30 cm) displayed similar anterior–posterior (pitch range; figure 7*b*,*e*) and medial–lateral (roll range; figure 7*c*,*f*) postural stability to each other, and significant differences (post hoc *t*‐test, *p* < 0.05) between tail lengths (15 and 30 cm) were only observed in 3 out of 56 post hoc pairwise comparisons. Furthermore, while there was significant variation in pitch range and roll range stability with speed (electronic supplementary material, tables S4–S6), for the tailed models there were not clear trends displaying a similar (less than 4° difference) pitch range and roll range across speeds (figure 7*b*–*f*; table 1, electronic supplementary material, table S1).

In contrast, for both myliobatid and NACA, tailless models differ significantly from both tailed models, presenting a higher pitch and roll range (post hoc *t*‐test: *p* < 0.05). For pitch range (anterior–posterior stability), this result is evidenced by a significant larger pitch range (myliobatid: 0 cm tail = 13.7 ± 0.4°, NACA: 0 cm tail = 17.6 ± 1.1°) compared with the tailed models (myliobatid: 15, 30 cm tail ≈ 8°, NACA: 15, 30 cm tails < 7°; table 1, electronic supplementary material, table S1). However, these differences were more pronounced at lower speeds (speed < 2.06 BL s^−1^). Roll range (medial–lateral stability) was the variable most affected by the tail length, especially at lower speeds where the roll ranges of models without tail were ± 25° (myliobatid: 0 cm tail = 27.08 ± 3.5°, NACA: 0 cm tails = 65.78 ± 8.7°; table 1 and electronic supplementary material, table S1), while the tailed models’ oscillations were less than ±5.5° (figure 7*c*,*f*; table 1 and electronic supplementary material, table S1). As flow speed increased, the roll range of the tailless models was reduced (and, therefore, the medial–lateral stability increased), and the tailless myliobatid model’s roll range was not significantly different to the tailed models once flow speed exceeded 2.4 BL s^−1^. However, the tailless NACA model maintained an elevated roll range compared with the tailed models until flow speeds exceeded 4.0 BL s^−1^. Furthermore, while the roll stability of the tailless myliobatid model increased rapidly with flow speed (figure 7*c*), the tailless NACA model’s stability increased more linearly, with the NACA model having a significantly higher roll range at flow speeds between 1.7 and 3.7 BL s^−1^ (figure 7*f*).

**Figure 8.**
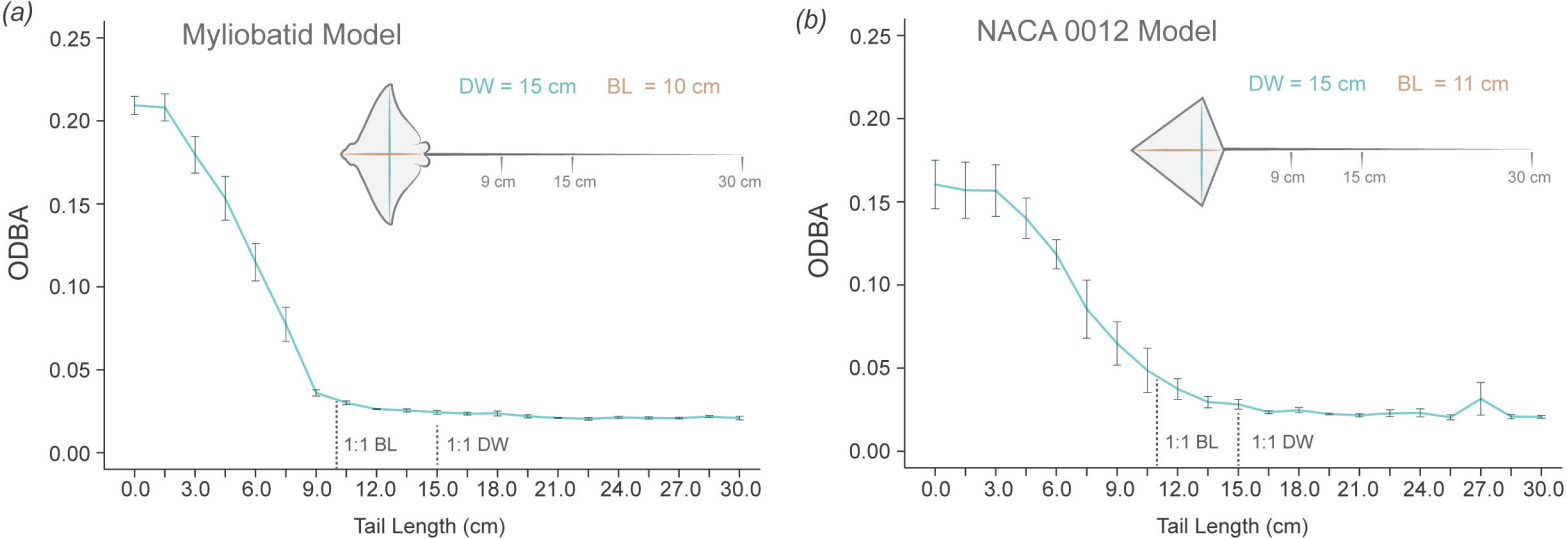
ODBA variation with progressive reduction in tail length. (*a*) ODBA values for the myliobatid model as the tail was progressively shortened by 1.5 cm. ODBA remained consistently below 0.05 g when the tail length was greater than 9 cm (0.9 BL). However, when the tail was shorter than 0.9 BL, ODBA drastically increased as the tail was shortened, reaching maximum values at 2.7 BL. (*b*) For the NACA 0012 model, ODBA remained relatively stable until the tail length dropped below 11 cm (1 BL), after which ODBA progressively increased, reaching the highest values with a tail length of 3 cm (0.27 BL). Note that BL differs for each model, being 10 cm for the myliobatid model and 11 cm for the NACA 0012 model. Error bars represent s.e. from the three trials conducted for each model. All experiments were performed at a flow speed of 1.4 BL s^−1^ (14 cm s^−1^). BL = body length, DW = disc width.

We also investigated the minimum tail length required to stabilize the models (figure 8). For the myliobatid model, tail lengths ranging from 3 times to 0.9 times the BL (STL 3–0.9 BL) effectively stabilized the model, with no observed differences among these lengths (figure 8*a*). However, when the STL was shorter than 0.9 BL, the myliobatid model began to destabilize critically, reflected in a rapid increase of ODBA with decreasing tail length (figure 8*a*). A similar trend was observed for the NACA model, although the increase in ODBA values was more gradual as the tail shortened (figure 8*b*). For the NACA model, the minimum STL to stabilize the model was 1.2 BL (12 cm tail length). Beyond this length, ODBA values progressively increased, peaking in the absence of a tail (figure 8*b*).

## 4. Discussion

In this study, we examine variation in the length and function of the long, slender tails across the four oscillatory families of Myliobatiformes and explore how these tails contribute to passive stability in models with pectoral fins held in an extended position. In this context, passive stability refers to the model’s inherent ability to resist and dampen undesired body motions without active muscular control or movement. Our results clearly indicate that models without tails are significantly less stable than models with tails, exhibiting greater roll (medial–lateral motion), pitch (anterior–posterior motion) and ODBA. Additionally, we identified that the minimum tail length required to stabilize the myliobatid-based model was 0.9 times the BL. Tails exceeding this threshold provided no additional stabilization, as models with tail lengths up to 3 times the BL or 2 times the DW—the maximum tested in this study—displayed similar levels of movement (figure 8). Interestingly, these findings align with the variation in relative tail length observed across myliobatid species (figure 3). The minimum tail length of 0.9 times BL is characteristic of the giant manta ray (*Mo. birostris*), whereas the maximum relative tail length of 4.6 times the BL is found in the spotted eagle ray (*A. narinari*). Notably, no myliobatid species examined had a tail length shorter than 0.9 BL. These differences in tail length suggest a dual role for tails in myliobatids, where (i) tails are playing a hydrodynamic role by passively stabilizing the body during gliding locomotion when the pectoral fins are not moving, and (ii) tails, especially longer lengths, are probably involved in other functions beyond stability, such as sensory or defensive roles [23].

### 4.1. Tails as body stabilizing structures

The distinctive body morphology of myliobatids, with their broad, tapered pectoral fins, generates thrust through lift-based mechanisms and achieves a high lift-to-drag ratio, enabling effective propulsion during the fins’ stroke cycles [8,10,12,19]. However, the function during locomotion of long whip-like tails, a distinctive feature of myliobatids, has often been overlooked.

Unlike other marine vertebrates, such as teleost fishes and sharks, myliobatids do not use their tails for propulsion. Instead, their tails are relatively rigid as they are supported by a long, mineralized rod of fused vertebrae (the caudal synarcual) that minimizes bending [23]. Still, the caudal synarcual allows for a certain degree of curvature (see electronic supplementary material, videos S1 and S2), suggesting that further analyses to understand the mechanical properties of the tail are needed. Additionally, during locomotion, myliobatids maintain the passively stiffened tail in a relatively straight position, as musculature is limited to the attachment point with the body, leaving the remainder of the tail almost devoid of muscles [23] (electronic supplementary material, videos S1 and S2).

**Figure 9.**
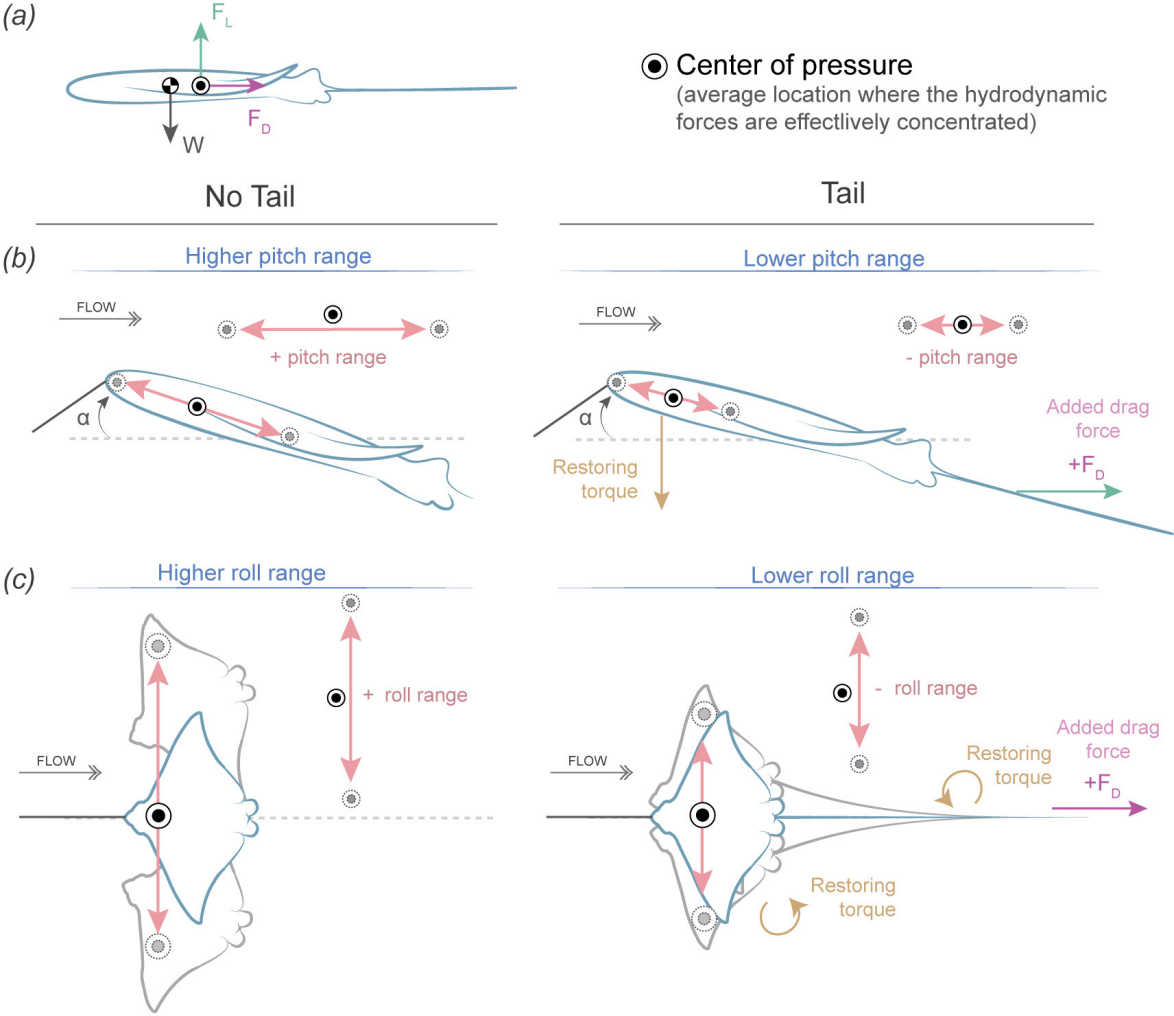
Schematic interpretation of the body stabilizing effect of the whip-like tail of myliobatids, illustrating the kite hypothesis of tail stability. (*a*) Simplified representation of the forces acting on a body gliding through flow with the pectoral fins extended. Forces include lift force (*F*_L_) and drag force (*F*_D_), which balance at the centre of pressure (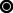), and weight (w), which is balanced at the centre of mass (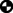). (*b*) Effects of the tail on pitch stability. We hypothesize that the tail helps stabilize the centre of gravity in anterior–posterior movement (pitch range). In the absence of a tail, the model exhibits a higher pitch range. However, a long, slender tail reduces pitch fluctuations by generating drag at the rear, damping forces and acting as a restoring torque, ultimately leading to a more stable posture. Since the tail is lightweight, it does not significantly alter the mass distribution or angle of attack but experiences friction drag as a function of its surface area which acts substantially posterior to the centre of mass, generating a large torque. (*c*) Effect of the tail on roll stability. The tail reduces roll range indirectly by limiting yawing and swaying movements around the centre of gravity. Models without tails experience greater roll fluctuations, whereas those with tails generate additional drag and damping forces, acting as a restoring torque that minimizes lateral–medial oscillations. To effectively reduce both pitch and roll range, the tail must be at least 0.9 BL in length.

The function of a tail in mediating body passive stability can be seen in analyses of flight by kites [35–37]. In kites, long and slender tails play a crucial aerodynamic role by passively increasing the stability of the kites while flying by reducing roll, yaw and pitch [35,37]. This resembles the effect of tail in our myliobatid and NACA models, where tails significatively decreased movement (ODBA) by reducing anterior–posterior (pitch) and medial–lateral (roll) rotations. As a result, we hypothesize that the same physical phenomena that apply to the tails in kites apply to myliobatids during gliding locomotion, an effect that we term the *kite hypothesis*. The physical phenomena generated by long and slender tails in kites are based on adding drag, which produces a restoring torque (figure 9) and, ultimately, stabilizes the centre of pressure (the location around which forces are balanced). While kites are often tethered with a bridle system in contrast to the nose tether used here, the effect of adding drag with an elongate tail posterior to the centre of mass and pressure on is similar.

Tails increase body stability by enhancing the system’s ability to resist deviations from its original orientation and return to equilibrium after a disturbance; therefore, we hypothesize that tails have an effect on the centre of pressure (figure 9). Without tails, the centre of pressure of our models has a larger range of anterior–posterior movement (which increases pitch range) and medial–-lateral movements (which increases roll range). In contrast, long and slender tails add drag posterior to both the centre of mass and pressure, a phenomenon known as drag-based stability [35,37,62]. By adding drag, tails act to damp oscillations, generating a restoring torque (rotational force) which reduces the range of movement of the centre of pressure and maintains it more centrally located within the body outline (figure 9). Reducing the range of movement of the centre of pressure reduces the response of the body to disturbances in roll and pitch. Technically, roll is a rotation around the drag vector, and thus additional drag from an elongate tail should not dampen and stabilize these movements. However, changes in the model’s roll, will alter the force balance acting on the model and thus induce changes in the model’s sway and roll, which were not measured in this study. However, an elongate tail should effectively dampen and stabilize these movements, and thus roll is probably stabilized indirectly through stabilization of yaw and sway. As the tail increases in length, it produces a more effective torque in restoring balance due to increased frictional drag force: total friction drag is a function of tail length and hence surface area. This effect was observed in our study, as tail lengths ranging from 0.9 to 3 times the BL (maximum length tested in our experiments) equally stabilized the models, with no significant difference in their effects on pitch and roll range, or ODBA. However, when the tail length dropped below 0.9 times BL, destabilization occurred (figure 8), increasing pitch and roll range and ODBA. This suggests that a tail shorter than this threshold does not experience sufficient drag and is therefore unable to generate sufficient restoring torque to stabilize the body, preventing it from damping oscillations and, therefore, not returning the model to its original position (figure 9).

In contrast, tails have minor effects on model posture. The tails of myliobatids, as well as the tails used in our study, are relatively light compared with the body of the animal (1.3% in real myliobatids and 2.3% in our models). Therefore, longer tails do not add significant weight that could affect the position of the centre of gravity (centre of mass), which is located anterior to the fin tips [6]. This is evident in our results, as models with larger tails displayed only minor differences in body posture and only at the slowest speeds. Lastly, myliobatid tails are relatively rigid, which limits their bending as rays swim and glide [23] (electronic supplementary material, videos S1 and S2).

To design our models to closely resemble real myliobatids, we conducted measurements of body density using preserved specimens from 10 myliobatid species (including eagle rays, pelagic eagle rays and cownose rays) to assess interspecific variation. Because specimens were preserved in ethanol, our measurements are approximate and rely on assumptions about tissue preservation, including shrinkage and complete fluid replacement. Despite these limitations, our estimated densities (mean ± s.d.: 1.08 ± 0.01 g ml^−1^) align with previous density measurements from fresh specimens of *R. bonasus* (1.02–1.05 g ml^−1^; [8,63]) supporting the validity of our approach. We found no significant differences in density among different myliobatid species, indicating that any of the myliobatid species measured are significantly heavier or lighter than others in terms of body density. These results provide new reference values for species that, due to their conservation status or limited specimen availability, are otherwise not available in the literature.

Finally, our experimental design presents both limitations and advantages. A tethered model differs from a free-swimming animal in several important ways, particularly in the location and distribution of forces. Tethering the model at the rostrum introduces a fixed point of force application that does not correspond to the centre of pressure, as it would in a freely swimming animal, which alters the free-body dynamics of the system. However, this effect does not invalidate our findings that the tail acts as a passive stabilizer by reducing oscillatory movements. While the towed protocol inherently restricts certain degrees of freedom (particularly pitch variation within a given flow speed due to the front attachment), it still allows meaningful comparison of pitch across models and speeds. Importantly, the set-up does not constrain roll or ODBA in those dimensions, allowing us to analyse these aspects of passive stability. The stabilizing effect of tails observed in this study, which dampens motions around the centre of pressure, is also observed in other systems like kites. Thus it would still apply to real animals, even if the force focal point differs.

### 4.2. Tail effect on body morphology

The effects of body morphology were tested by comparing a model based on a myliobatid ray body with a model based on a classic NACA 0012 aerofoil body. Both tail lengths (15 and 30 cm) equally stabilized both models, resulting in similar ODBA values across all flow speeds. This indicates that light, long and slender tails are crucial structures and function to increase stability similarly in different body shapes. However, without tails, models behave differently, which demonstrates the effect of body morphology. Without tails, the NACA model showed lower ODBA values than the myliobatid model, indicating that the NACA shape is more stable than a myliobatid triangular shape. However, flow speed had different impacts on stabilizing both models without tails. While the tailless myliobatid model stabilized at flow speeds higher than approximately 2.4 BL s^−1^, showing similar ODBA values to models with tails, the tailless NACA model always maintained higher ODBA values than models without tails across all speeds. This indicates that the myliobatid morphology is able to stabilize itself at high flow speeds, while the NACA model cannot. In addition, the minimum tail length required to stabilize the models differed between NACA and myliobatid body models. The minimum tail length to stabilize the myliobatid model was 0.9 BL: at tail lengths less than this value, the model became unstable. For NACA body model, instability began at tail lengths less than 1.2 BL (figure 8).

### 4.3. The role of tail length in myliobatid biology

In this study, we found interesting correspondences between our experimental results on body models and measurements of tail length in myliobatid rays. *Mobula birostris* presented the shortest tails relative to BL, with tail measurements ranging from 0.9 to 1.0 BL, while all other myliobatids presented tails longer than 1.1 BL. Interestingly, a minimum tail length of 0.9 BL was also required to experimentally stabilize the myliobatid model (figure 8). Although only two specimens of *Mo. birostris* were available, due to the difficulty of accessing this species, the absence of tails shorter than this threshold across all measured myliobatid species suggests that tail length plays a role in maintaining body stability during gliding, when active propulsion is absent.

Our experimental tests also show that tails greatly increase body stability at speeds between 1 and 2.5 BL s^−1^. This result agrees with the speed range recorded for swimming and gliding myliobatids, as reported by [10,64–66] who noted a speed range of 0.2–1.25 BL s^−1^ for *Mo. birostris*. The fact that the minimum tail length required to stabilize the model (0.9BL) matches the shortest tail length observed in real myliobatid species suggests that the tail may play a similar stabilizing role *in vivo*. This passive contribution could reduce the need for continuous muscular adjustments during locomotion, potentially lowering energetic costs during gliding or swimming.

In contrast, tails longer than 0.9 BL did not provide additional stability, suggesting that in species with tails exceeding this threshold, tails may be involved in additional functions. This idea is supported by the observed variation in relative tail length across myliobatid species and throughout ontogeny (figure 3). Although myliobatids are described as ‘pelagic rays’, migratory species able to efficiently swim long distances and over long periods of time, species differ considerably in ecology and lifestyle. Species from the families Aetobatidae (e.g. the spotted eagle ray *A. narinari*), Rhinopteridae (e.g. the cownose ray *R. bonasus*) and Myliobatidae (bat eagle ray *My. californica*) are benthopelagic predators, since they mainly predate on molluscs buried in the sand [67–72]. In contrast, devil and manta rays (family Mobulidae) are filter-feeders that feed on plankton that live suspended in the water column (and are specialized to detect and predate on them using their cephalic lobes) and, therefore, are not associated with the sea-bottom [73–76]. However, although devil and manta rays present similar feeding behaviour, they differ in size; while manta rays (*Mo. birostris, Mo. alfredi* and *Mo. yarae*) are some of the largest fishes on the ocean, devil rays are similar in size to other myliobatids [7].

Our results indicate that tail length correlates with body size, regardless of feeding ecology (figure 3). While larger specimens have the shortest tails, smaller species, such as cownose, eagles and devil rays, have similar relative tail lengths. These findings suggest a possible correlation between tail length and the capacity to manoeuvre (defined as the ability to change position, direction and orientation while maintaining stability). Smaller rays are more agile and capable of rapid turns compared with manta rays, and relatively larger tails may help in maintaining stability during rapid turns [11,77]. Additionally, myliobatid tails may be related to predator avoidance. Smaller rays are also more vulnerable to predators than larger rays (e.g. sand sharks, hammerheads and killer whales are known predators of several myliobatid species) [74,78,79]. The mechanosensory system of the tail—which contains an extensive lateral line [23]—may play a crucial role in improving predator detection: longer tails extend the spatial range for detecting predators approaching from behind, giving rays sufficient time to escape. Previous work has shown that rays initiate an escape response when a sudden water movement is produced near the tail [23]. Finally, the tail may play a role in inter-individual communication and may facilitate social interactions during the formation of large schools (common in myliobatid species) [80–82] and mating.

## Data Availability

Biologger raw data and specimen measurement values are publicly available in the Mendeley Repository [83]. Supplementary material is available online [84].
